# Clinical Outcomes and Astigmatism Vector Analysis of High Toricity Visian ICL

**DOI:** 10.22336/rjo.2026.15

**Published:** 2026

**Authors:** Silvia Victoria Prodescu, Paul Filip Curcă, Cătălina Ioana Tătaru, Călin Petru Tătaru

**Affiliations:** 1 Department of Ophthalmology, Doctoral School, “Carol Davila” University of Medicine and Pharmacy, Bucharest, Romania; 2 Department of Ophthalmology, Alcor Clinic, Bucharest, Romania; 3 Department of Ophthalmology, “Dr. Carol Davila” Central Military Emergency University Hospital, Bucharest, Romania; 4 “Carol Davila” University of Medicine and Pharmacy, Bucharest, Romania; 5 Department of Ophthalmology, “Prof. Dr. Mircea Olteanu” Clinical Institute for Ophthalmological Emergencies, Bucharest, Romania

**Keywords:** toric implantable collamer lens, high astigmatism, vector analysis, refractive outcomes, postoperative vault, ICL = implantable collamer lens, UDVA = uncorrected distance visual acuity, CDVA = corrected distance visual acuity, logMAR = logarithm of the minimum angle of resolution, IOP = intraocular pressure, ECD = endothelial cell density, WTW = white-to-white, AQD = aqueous depth, ACD = anterior chamber depth, ACA = anterior chamber angle, SEQ = spherical equivalent, AL = axial length, CCT = central corneal thickness, K1 = flat keratometry, K2 = steep keratometry, D = diopter, SD = standard deviation, IQR = interquartile range, N = number of, Nd:YAG = neodymium-doped yttrium aluminum garnet (laser), WTR = with-the-rule, ATR = against-the-rule, SI = safety index, EI = efficacy index, TIA = target-induced astigmatism, SIA = surgically induced astigmatism, DV = difference vector, CI = correction index, IOS = index of success, ME = magnitude of error, AE = angle of error, OCT = optical coherence tomography, SS-OCT = Swept-source optical coherence tomography, AS SS-OCT = Anterior segment swept-source optical coherence tomography

## Abstract

**Objective:**

To evaluate visual, refractive, and astigmatism vector outcomes following toric implantable collamer lens (ICL) implantation in eyes with high astigmatism.

**Methods:**

A retrospective real-world study was conducted on 25 eyes (16 patients) undergoing toric ICL implantation. Visual acuity, refractive outcomes, and astigmatic correction were assessed at 1 year postoperatively. Vector analysis was performed according to current reporting standards. Postoperative vault and its association with biometric parameters were also analyzed.

**Results:**

Mean UDVA improved from counting fingers to 0.16 ± 0.16 logMAR, and CDVA from 0.26 ± 0.21 to 0.09 ± 0.11 logMAR. The mean residual spherical equivalent was −0.56 ± 0.77D, and the mean residual astigmatism was 0.99 ± 0.72D. Vector analysis showed a correction index of 0.94 ± 0.25, indicating effective and proportional correction. The mean vault measured 615.5 ± 192.3 µm, and was significantly associated with ICL size (p=0.002), whereas no significant correlation was found with white-to-white measurements (ρ=0.15).

**Discussion:**

Slightly lower refractive predictability may reflect the inclusion of higher astigmatism eyes and limitations of available ICL toricity, while the lack of correlation between white-to-white measurements and vault highlights the role of internal anatomical factors in ICL sizing.

**Conclusions:**

Toric ICL implantation is safe and effective for high astigmatism. Vector analysis confirms accurate astigmatic correction, while postoperative vault is primarily influenced by ICL size rather than external biometric measurements. These findings support sizing strategies based on internal ocular anatomy and offer insights into the astigmatic outcomes in a cohort with high astigmatism, a subgroup underrepresented in current literature.

## Introduction

Accurate correction of high astigmatism remains challenging. While corneal refractive surgery can achieve excellent results in selected eyes, higher cylindrical corrections are more likely to approach biomechanical limits, induce higher-order aberrations, and demonstrate reduced predictability, particularly when combined with high spherical ametropia [1-4].

Posterior chamber phakic intraocular lenses have emerged as an alternative that preserves corneal architecture [5,6]. The Visian toric implantable collamer lens (ICL) enables correction of a wide range of sphero-cylindrical errors. It is particularly attractive in younger patients with high ametropia in whom preserving accommodation is desirable. Reported advantages include reversibility, broad dioptric availability, rapid visual rehabilitation, and favorable optical quality [6-9]. The introduction of a central port design has further improved the safety profile by facilitating the flow of aqueous humor [9].

Nevertheless, phakic IOL implantation still carries potential risks, including endothelial cell loss, intraocular pressure elevation [10], cataract formation [8], and rotational instability of toric lenses [11]. Postoperative vault remains a critical parameter closely linked to lens sizing and anterior segment anatomy [12,13], and optimal sizing strategies continue to be investigated, with known variability among white-to-white measurement techniques [14,15] complicating preoperative planning.

Although toric ICL outcomes have been widely reported, relatively few studies focus specifically on eyes with higher degrees of astigmatism, and standardized vector analysis [16-18] of astigmatic correction is not consistently applied. At the same time, the relationship between preoperative biometric parameters and postoperative vault remains incompletely defined [12,13,19]. This subgroup of high astigmatism refractive surgery cases treated with toric ICL is underrepresented in the literature, as young patients are often directed toward corneal procedures (if corneal parameters permit surgical safety) and older patients toward lens-based surgery.

The present study aims to evaluate visual and refractive outcomes, including standardized vector analysis of astigmatic correction [18,20] and postoperative vault analysis, following toric ICL implantation in eyes with preoperative refractive cylinder greater than 2.00 D, in a real-world clinical setting with 1-year follow-up.

## Materials and methods

### Study design and ethics

This study included eyes with preoperative refractive astigmatism ≥ 2.00 D that underwent toric Implantable Collamer Lens (ICL; STAAR Surgical, Lake Forest, CA, USA) implantation performed by a single experienced anterior segment surgeon in a private practice setting in Bucharest, Romania, between April 2021 and April 2025. Data were prospectively collected and retrospectively analyzed as part of a doctoral research project conducted at “Carol Davila” University of Medicine and Pharmacy, Bucharest. The study adhered to the tenets of the Declaration of Helsinki, and all patients provided written informed consent. Ethical approval (number 1582/14.04.2026) was obtained from the independent ethics committee (the Local Ethics Committee for Scientific Research of the “Prof. Dr. Mircea Olteanu” Clinical Institute for Ophthalmological Emergencies, Bucharest).

### Study population

A total of 25 eyes of 16 patients were included. Inclusion criteria were age ≥ 18 years, refractive astigmatism ≥ 2.00 D, and eligibility for toric ICL implantation, as determined by clinical assessment and patient preference. Cases were not randomized. Exclusion criteria included refusal to participate, incomplete medical records, loss to follow-up before 1 year postoperatively, anterior chamber angle or endothelial cell density below safety thresholds, glaucoma, keratoconus, and cases planned for sequential bioptics procedures. Eyes with pre-existing amblyopia or strabismus were not excluded, as these conditions are commonly encountered in the high-ametropia population. The target refraction was emmetropia in all cases; however, the predicted residual refractive error varied due to the discrete power steps available for ICL manufacturing and the limitations of the available toric power.

### Preoperative assessment

All patients underwent a comprehensive preoperative ophthalmic examination, including uncorrected and corrected distance visual acuity (UDVA, CDVA), intraocular pressure (IOP), manifest and cycloplegic refraction, slit-lamp examination, ophthalmoscopy, and endothelial cell density (ECD). Biometric and tomographic measurements were obtained using ANTERION (Heidelberg Engineering, Heidelberg, Germany) and Allegro Oculyzer Wavelight (Alcon Laboratories, Inc., Fort Worth, TX, USA), supplemented by autorefractometry. White-to-white (WTW) diameter was measured using ANTERION and manually with a sterile Castroviejo caliper under operating microscope magnification. Aqueous depth (AQD) was measured from the corneal endothelium to the anterior lens capsule using ANTERION. Anterior chamber angle was assessed preoperatively and categorized as ≤ 30°, 30-45°, or ≥ 45°. Additional investigations, including optical coherence tomography, were performed as clinically indicated.

### ICL calculation and lens selection

Preoperative toric ICL planning was based on manifest and cycloplegic refraction, keratometry, central corneal thickness, aqueous depth, WTW distance, ECD, and anterior chamber angle. Keratometric and anterior segment measurements were reviewed across available devices and reconciled with autorefractometry data when discrepancies arose. The final values entered into the toric ICL calculator were selected based on concordance across multimodal measurements and clinical judgment. ICL power, cylindrical correction, size, and alignment axis were determined using the manufacturer’s online calculation and ordering system (OCOS; STAAR Surgical, Monrovia, CA, USA). The implanted lenses included toric myopic (VTICM0, V4c; VTICM5, V5) and toric hyperopic (VTICH) models in sizes 12.1 mm, 12.6 mm, and 13.2 mm (manufactured by STAAR Surgical, Nidau, Switzerland).

### Surgical technique

**Fig. 1:**
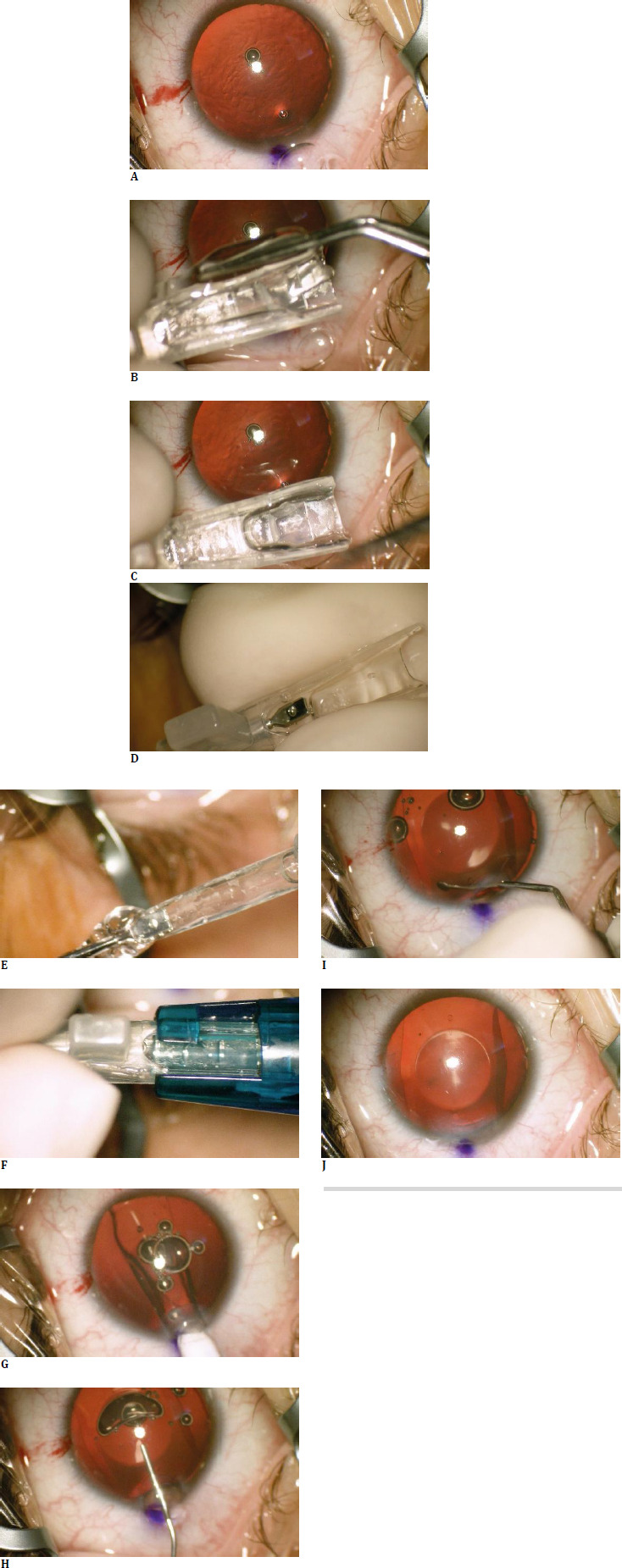
Surgical Steps for Toric ICL Implantation. **A**. Injection of viscoelastic surgical device (OVD). **B**. Folding the ICL into the cartridge. **C**. Folded ICL in the cartridge. **D**. Advancing the folded ICL towards the front of the cartridge. **E**. Final ICL position within the cartridge before implantation. **F**. Locking the cartridge within the injector. **G**. ICL Implantation. **H**. Injection of OVD before any further positioning maneuvers. **I**. Positioning the haptics in the sulcus using a specialized spatula. **J**. Final aspect with Visian ICL positioned on axis (surgical marker visible at limbus with purple, toric ICL markings visible at the edge of the round optical zone, above and below central intraoperative light source reflex)

All surgeries were performed in the same operating room by the same surgeon. The intended alignment axis was marked preoperatively at the slit lamp. Under topical anesthesia, a temporal 3 mm clear corneal main incision and a secondary paracentesis were created, followed by injection of ophthalmic viscosurgical device, ICL implantation, alignment to the intended axis, and complete removal of viscoelastic material (**Fig. 1A-J**). Wound integrity was ensured by hydrosuture. Peripheral laser iridotomy was performed preoperatively with an Nd:YAG laser in two eyes receiving hyperopic toric ICLs; all other eyes received ICLs with a central port (KS-AquaPORT®) design, which does not require iridotomy. Postoperative treatment consisted of topical antibiotic-steroid combination drops and preservative-free artificial tears. No intraoperative complications occurred.

### Postoperative follow-up

**Fig. 2:**
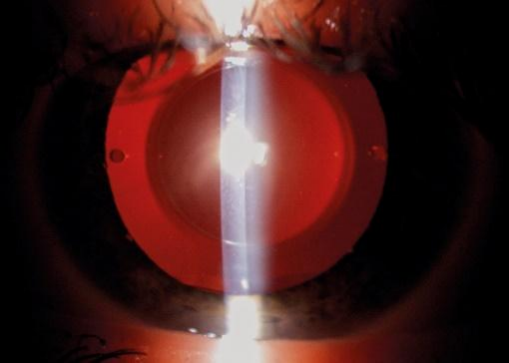
Postoperative aspect of Toric Visian ICL. Toric Markings are placed on axis close to 180° and are perpendicular to the light projected by the slit-lamp

**Fig. 3:**
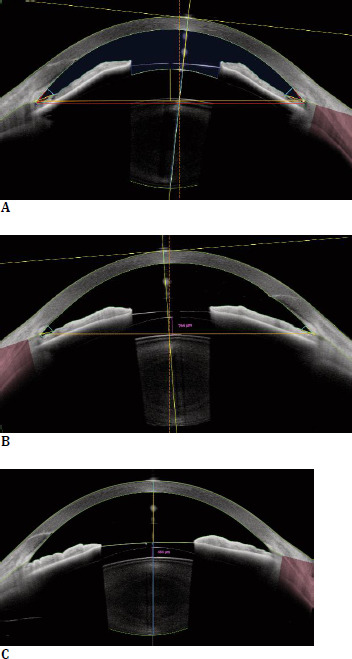
Vault imaging. Vault was categorized as low (< 250 µm), optimal (250-750 µm), or high (> 750 µm). **A**. High vault (1400 µm), **B**. Near-optimal vault of 764 µm, and **C**. A lower vault of 464 µm, still within normal range. Vault was measured as the perpendicular distance between the posterior ICL surface and the anterior lens capsule using AS SS-OCT ANTERION (Heidelberg Engineering, Heidelberg, Germany)

Postoperative examinations were performed at 3-5 days, 1 month, and approximately 1 year (range 11-14 months) after surgery. The primary analysis was conducted on the 1-year data. Evaluations included UDVA, CDVA, manifest refraction, IOP, slit-lamp examination (**Fig. 2**), and assessment of complications. Postoperative vault was measured systematically at 1 month using the ANTERION metrics module, defined as the distance between the posterior surface of the ICL and the anterior lens capsule. Vault was categorized as low (< 250 µm), optimal (250-750 µm), or high (> 750 µm) (**Fig. 3A-C**).

### Outcome measures and data analysis

Visual acuity was recorded in decimal notation and converted to the logarithm of the minimum angle of resolution (logMAR) for statistical analysis. Counting fingers vision was assigned a logMAR value of 2.0. For the construction of standard refractive surgery outcome graphs, logMAR values were converted to Snellen equivalents. Spherical equivalent (SEQ) was calculated as sphere + cylinder/2. Preoperative astigmatism orientation was classified as with-the-rule (WTR; steep meridian axis 60-120°), against-the-rule (ATR; steep meridian axis 0-30° or 150-180°), or oblique (steep meridian axis 31-59° or 121-149°). The astigmatism type was classified as myopic, hyperopic, or mixed based on the signs of the principal meridians. The efficacy index was defined as the ratio of mean postoperative UDVA to mean preoperative CDVA (in decimal), and the safety index as the ratio of mean postoperative CDVA to mean preoperative CDVA (in decimal).

### Statistical analysis

Statistical analysis was performed on an eye-based dataset. Data were recorded in Microsoft Excel (Microsoft Corporation, Redmond, WA, USA) and analyzed using Minitab (version 20, Minitab LLC, State College, PA, USA). Continuous variables were presented as mean ± standard deviation (range) unless otherwise specified, and categorical variables were reported as frequencies and percentages (n - %). Angular variables with non-normal distributions were reported as medians (interquartile ranges). A p-value < 0.05 was considered statistically significant. Agreement between device-measured and manual WTW was assessed using Bland-Altman analysis, with calculation of mean difference and 95% limits of agreement. Normality of paired differences was evaluated using the Anderson-Darling test. Paired comparisons were performed using paired t-tests. The association between postoperative vault and ICL size was assessed using the Kruskal-Wallis test. Correlation between WTW and vault was evaluated using Spearman’s rank correlation with 95% confidence intervals.

### Astigmatism vector analysis

Vector analysis of astigmatic correction was performed according to the Alpins method [20] using AstigMETRICS [21] and mEYEstro [22]. Graphical representations, including double-angle plots, were generated using the ASCRS Astigmatism Double Angle Plot Tool (available at https://www.ascrs.org/en/tools/astigmatism-double-angle-plot-tool as described by Abulafia et al. [23]). For each eye, the following vectors and indices were calculated: target-induced astigmatism (TIA), surgically induced astigmatism (SIA), difference vector (DV), correction index (CI), index of success (IOS), magnitude of error (ME), and angle of error (AE). Manifest refractions were transposed to plus-cylinder notation before vector and graphical analyses. Refractive astigmatism was recorded at the spectacle plane (vertex distance 12 mm) and converted to the corneal plane for vector metrics. Preoperative corneal astigmatism for the double-angle plot input was derived from anterior keratometry (K2-K1) measured with ANTERION. Residual astigmatism was further summarized using centroid analysis derived from double-angle vector components.

## Results

### Patient Demographics

A total of 25 eyes from 16 patients were included in the analysis and are summarized in **Table 1**. The mean age at surgery was 26.8 ± 5.4 years (range 20-39). Eight patients (50%) were female. Bilateral implantation was performed in 9 patients (56.3%). Among the treated eyes, 12 (48.0%) were right eyes, and 13 (52.0%) were left eyes. All included eyes completed the 1-year follow-up.

**Table 1:** Demographic characteristics of the study population

Metric	Value
N patients	16
N eyes	25
Mean Age (years) ± SD (range)	26.8 ± 5.4 (20-39)
**Sex**	**N (%)**
Female	8 (50.0%)
Male	8 (50.0%)
**Laterality**	**N (%)**
Right Eye	12 (48.0%)
Left Eye	13 (52.0%)
N bilateral patients (%)	9 (56.3%)

N = number of patients or eyes, as specified; SD = standard deviation.

### Baseline characteristics and preoperative data

**Table 2** presents the baseline visual, refractive, and biometric characteristics of the study cohort. Preoperatively, all eyes had uncorrected distance visual acuity (UDVA) of counting fingers. The mean CDVA was 0.26 ± 0.21 logMAR. The mean manifest spherical equivalent (SEQ) was -11.26 ± 6.62 (from -17.38 to 8.50) D, with a mean refractive cylinder (absolute value) of 3.72 ± 1.28 (2.25 to 8.00) D. Astigmatism was predominantly with-the-rule (88%), and most eyes presented myopic astigmatism (88%). Mean keratometry values were 42.91 ± 2.17 D for K1 and 46.03 ± 2.09 D for K2, corresponding to a mean anterior corneal astigmatism of 3.13 ± 1.22 D. The mean central corneal thickness was 532 ± 29 µm, and the mean axial length (AL) was 27.35 ± 2.42 mm for the 22 eyes with available AL data.

**Table 2:** Preoperative Visual and Refractive Characteristics

Variable	Value
UDVA (logMAR)	2.00 (all eyes; counting fingers)
CDVA (logMAR)	0.26 ± 0.21
Manifest refractive Sphere (D)	-9.40 ± 6.92 (-15.00 to 10.75)
Manifest refractive cylinder (D, absolute)	3.72 ± 1.27 (2.25 to 8.00)
Spherical Equivalent (D)	-11.26 ± 6.62 (-17.38 to 8.50)
***Astigmatism Orientation***	
WTR (with-the-rule)	22 (88.0%)
ATR (against-the-rule)	1 (4.0%)
Oblique	2 (8.0%)
***Astigmatism Type***	
Myopic	22 (88.0%)
Mixed	1 (4.0%)
Hyperopic	2 (8.0%)
**Biometric and Corneal Characteristics**	
K1 (flat keratometry, D)	42.91 ± 2.17
K2 (steep keratometry, D)	46.03 ± 2.09
Anterior corneal astigmatism (D)	3.13 ± 1.22
WTW (mm, ANTERION - measured)	11.96 ± 0.35
WTW (mm, measured with manual caliper)	11.67 ± 0.36
Mean paired difference (device WTW vs. manual WTW)	0.26 (95% confidence interval 0.17 to 0.35 mm), p<0.001*
AQD (mm)	3.05 ± 0.30
CCT (µm)	532 ± 29
AL (mm)	27.35 ± 2.42 (n = 22)
ECD (cells/mm^2^)	2703 ± 309
**Anterior Chamber Angle (ACA, degrees)**	
≤ 30°	2 (8.0%)
30°-45°	21 (84.0%)
≥45°	2 (8.0%)

Data are presented as mean ± standard deviation (range) or number (percentage), as appropriate. N = 25 eyes unless otherwise specified. ^*^Paired t-test UDVA = uncorrected distance visual acuity; CDVA = corrected distance visual acuity; D = diopters; logMAR = Logarithm of the Minimum Angle of Resolution; WTW = white-to-white; AQD = aqueous depth; CCT = central corneal thickness; AL = axial length; ACA = anterior chamber angle; ECD = endothelial cell density.

Biometric parameters relevant for ICL sizing calculations and safety included: white-to-white diameter (WTW), aqueous depth (AQD), endothelial cell density (ECD), and the anterior chamber angle (ACA). Mean device-measured WTW was 11.96 ± 0.35 mm compared to 11.67 ± 0.36 mm by manual caliper. Mean ACD, measured from the corneal endothelium to the anterior lens capsule plane, was 3.05 ± 0.30 mm, and mean ECD was 2703 ± 309 cells/mm^2^. Anterior chamber angle measurements showed that most eyes (84.0%) had angles between 30° and 45°, with only 2 eyes each in the ≤30° and ≥45° categories.

### WTW diameter measurements

**Fig. 4:**
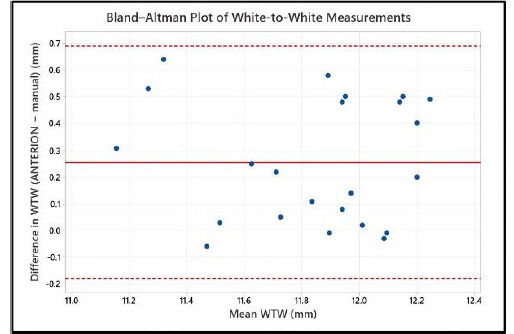
Bland-Altman Plot showing agreement between device-measured and manual white-to-white (WTW) values. The solid line represents the mean difference (bias) between ANTERION-derived and manual caliper WTW (white-to-white) measurement; dashed lines represent the 95% limits of agreement (-0.18 to 0.69 mm)

Paired comparison showed that device-measured WTW values were significantly higher than manual caliper measurements, with a mean difference of 0.26 mm (95% confidence interval 0.17 to 0.35 mm, p < 0.001). Bland-Altman analysis (**Fig. 4**) demonstrated a mean difference of 0.26 mm between measurement methods, with 95% limits of agreement ranging from −0.18 to 0.69 mm. No evidence of proportional bias was observed.

### Surgical data

**Table 3** presents the surgical parameters. The mean implanted ICL spherical power was **−**13.70 ± 7.07, and the mean cylindrical power was 3.72 ± 1.27 D. The median ICL toric correction axis was 89° (IQR 73.0°-92.5°), and the median planned alignment axis was 169° (IQR 5.5°-175.5°). The most frequently implanted lens size was 13.2 mm (44%), followed by 12.6 mm (32%) and 12.1 mm (24%). The target postoperative refraction, as predicted by the ICL calculation software, was −0.39 ± 0.65 D for sphere and 0.46 ± 0.82 D for cylinder, with as near to emmetropia as possible as the intended refraction in all cases. The residual predicted refraction likely reflected the discrete, limited power steps available in ICL manufacturing, which did not permit complete neutralization of the preoperative refractive error in every case.

**Table 3:** Surgical Parameters and ICL Characteristics (n=25 eyes)

Variable	Value
ICL spherical power (D)	-13.70 ± 7.07 (-18.00 to +8.50)
ICL cylindrical power (D)	3.72 ± 1.27 (2.00-6.00)
ICL cylinder axis (°)	89.0 [73.0-92.5]
**Intended alignment axis (°)**	169.0 [5.5-175.5]
**ICL size, n (%)**	
12.1 mm	6 (24.0%)
12.6 mm	8 (32.0%)
13.2 mm	11 (44.0%)
**Target sphere (D)**	−0.39 ± 0.65 (-2.17 to 0.24)
**Target cylinder (D)**	0.46 ± 0.82 (0.02 to 3.85)
**Target axis (°)**	90.0 [81.5-100.5]

Continuous variables reported as mean ± SD (range). Categorical variables reported as n (%). Angular variables reported as median (IQR). ICL = implantable collamer lens; D = diopters; mm = millimeters; SD = standard deviation.

### Visual and refractive outcomes

**Fig. 5:**
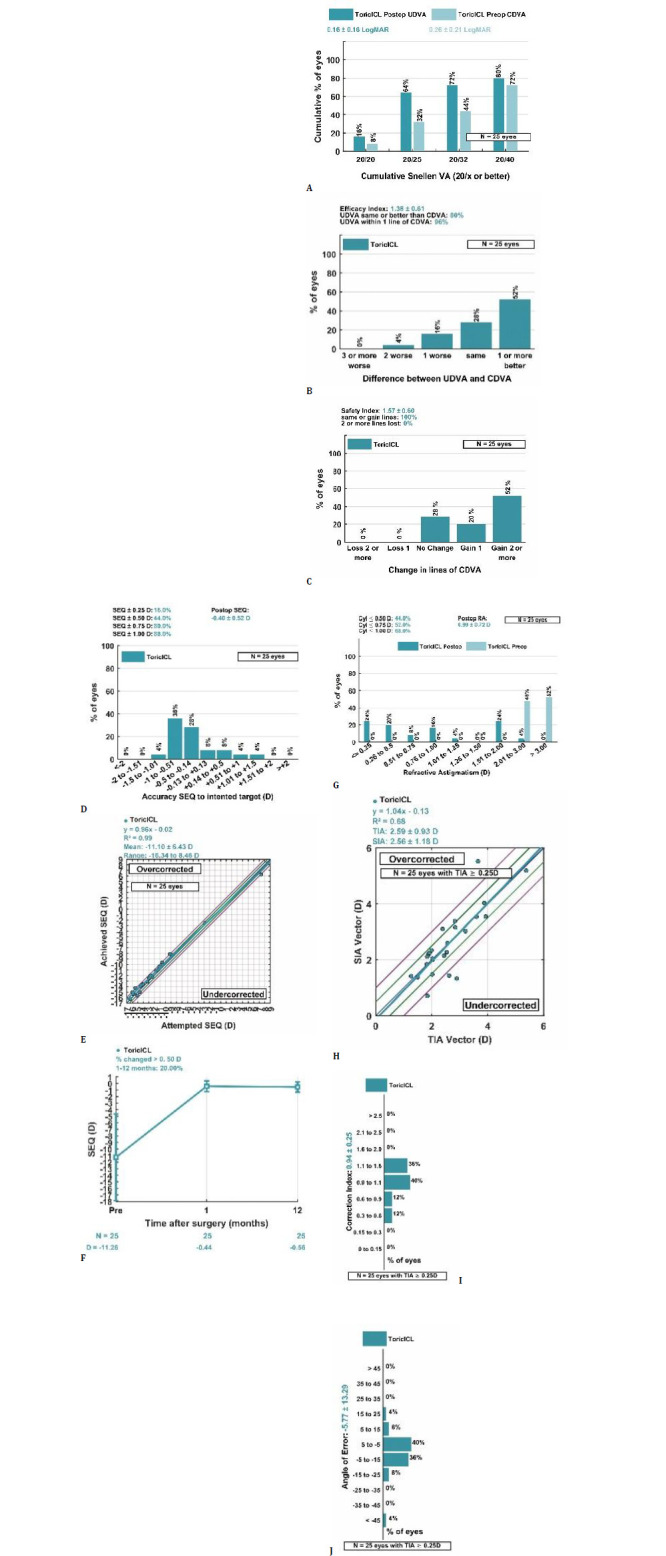
Standard visual and refractive outcomes following toric ICL implantation at 1 year postoperative. **A**. Cumulative uncorrected distance visual acuity (UDVA). **B**. Distribution of postoperative UDVA relative to corrected distance visual acuity (CDVA). **C**. Changes in CDVA lines from preoperative to postoperative values. **D**. Distribution of Spherical Equivalent (SEQ) accuracy relative to the intended target. **E**. Attempted versus achieved SEQ showing strong linear correlation. **F**. Stability of SEQ over time from 1 month to 1 year postoperative. **G**. Distribution of residual refractive astigmatism. **H**. Relationship between Target-Induced Astigmatism (TIA) and Surgically-Induced Astigmatism (SIA). **I**. Distribution of Correction Index (CI). **J**. Distribution of Angle of Error (AE). Standard graphs were generated from the original data using mEYEstro software, in accordance with current reporting guidelines for refractive surgery outcomes [22]

Primary analysis of the visual and refractive outcomes was conducted on the 1-year follow-up data (n=25 eyes). Results are presented in **Table 4** and illustrated in **Fig. 5A-J**.

**Table 4:** Postoperative Visual and Refractive Outcomes at 1 Year (n=25 eyes)

Variable	Value
Manifest sphere (D)	-0.15 ± 0.65 (-2.50 to 0.50)
Manifest cylinder (absolute, D)	0.99 ± 0.72 (0.25 to 2.75)
Spherical equivalent (D)	-0.56 ± 0.77 (-2.75 to 1.50)
UDVA (logMAR)	0.16 ± 0.16 (0.00 to 0.52)
CDVA (logMAR)	0.09 ± 0.11 (0.00 to 0.40)

Data are presented as mean ± standard deviation (range). UDVA = uncorrected distance visual acuity; CDVA = corrected distance visual acuity; logMAR = logarithm of the minimum angle of resolution; D = diopters.

At 1 year postoperatively, the mean UDVA improved from counting fingers (logMAR 2.00) to 0.16 ± 0.16 logMAR, and the mean CDVA improved from 0.26 ± 0.21 to 0.09 ± 0.11 logMAR (**Table 4**). Visual acuity improvements yielded an efficacy index of 1.38 ± 0.61, with postoperative UDVA equal to or better than preoperative CDVA in 80% of eyes and within one line in 96% (**Fig. 5B**). Additionally, the safety index was 1.57 ± 0.60; no eye lost two or more lines of CDVA, while 72% gained one or more lines (**Fig. 5C**).

The mean postoperative SEQ was −0.56 ± 0.77 D. SEQ predictability was within ±0.50 D of the target in 44% and within ±1.00 D in 88% of eyes (**Fig. 5D**). The attempted versus achieved SEQ plot demonstrated excellent correlation (R^2^ = 0.99, slope = 0.96), with a slight trend toward undercorrection in higher myopic corrections (**Fig. 5E**).

Refractive stability was maintained between 1 and 12 months (with only a slight myopic shift in mean SEQ from -0.44 D at 1 month to -0.56 D at 12 months, a mean clinically negligible change of -0.12 D), with 20% of eyes showing a change of more than 0.50 D in SEQ over this period (**Fig. 5F**).

Regarding astigmatic correction, the mean postoperative refractive cylinder was 0.99 ± 0.72 D, with 44% of eyes achieving ≤ 0.50 D and 68% achieving ≤ 1.00 D (**Fig. 5G**). To further characterize astigmatic correction following toric ICL implantation beyond scalar residual cylinder metrics, vector analysis according to the Alpins methodology [20] was conducted on the primary 1-year endpoint results.

### Vector analysis of astigmatic correction

Standard vectors for astigmatism analysis are presented in **Table 5**, while graphical representations of the outcomes are shown in the standard graphs in **Fig. 5 H-J**.

Vector analysis showed a mean TIA of 2.59 ± 0.93 D and a mean SIA of 2.56 ± 1.18 D, resulting in a mean difference vector (DV) of 0.84 ± 0.63 D and a correction index (CI) of 0.94 ± 0.25 (**Fig. 5 I**). The mean magnitude of error (ME) was −0.03 ± 0.67 D, with 72% of eyes within ±0.50 D and 84% within ±1.00 D, indicating no clinically significant systematic over- or undercorrection of astigmatism magnitude (**Table 5**).

**Table 5:** Astigmatism Vector Analysis at 1 year (N = 25 eyes with TIA ≥ 0.25 D)

Variable	
TIA (D)	2.59 ± 0.93
SIA (D)	2.56 ± 1.18
DV (D)	0.84 ± 0.63
CI	0.94 ± 0.25
IOS	0.35 ± 0.27
ME (D)	-0.03 ± 0.67
AE (°)	-5.77 ± 13.29
% ME within ±0.50 D	72%
% ME within ±1.00 D	84%
% with |AE| within 15°	84%
% with AE > 15°	4%
% with AE < -15°	12%

Data are presented as mean ± standard deviation or percentage, as appropriate. TIA = target-induced astigmatism; SIA = surgically induced astigmatism; DV = difference vector; CI = correction index; IOS = index of success; ME = magnitude of error; AE = angle of error; D = diopters. Vector analysis performed using the Alpins method^20^. Result generated from original statistical data with astigMETRICS software [21]

The relationship between TIA and SIA (**Fig. 5H**) demonstrated a strong linear correlation, with a slope of 1.04 and an R^2^ of 0.68, indicating good proportionality between intended and achieved astigmatic correction. The slope close to unity suggested an accurate magnitude of astigmatic correction, although some variability remained, as reflected by the R^2^ value. The index of success (IOS) was 0.35 ± 0.27.

Regarding astigmatism orientation, the mean angle of error (AE) was −5.77 ± 13.29° (**Table 5**) with 84% of eyes within ±15° of the intended axis (histogram in **Fig. 5J**). Among eyes outside this range, 12% had an angle of error less than -15° and 4% greater than 15°, suggesting a mild counterclockwise rotational tendency in a minority of cases.

**Fig. 6:**
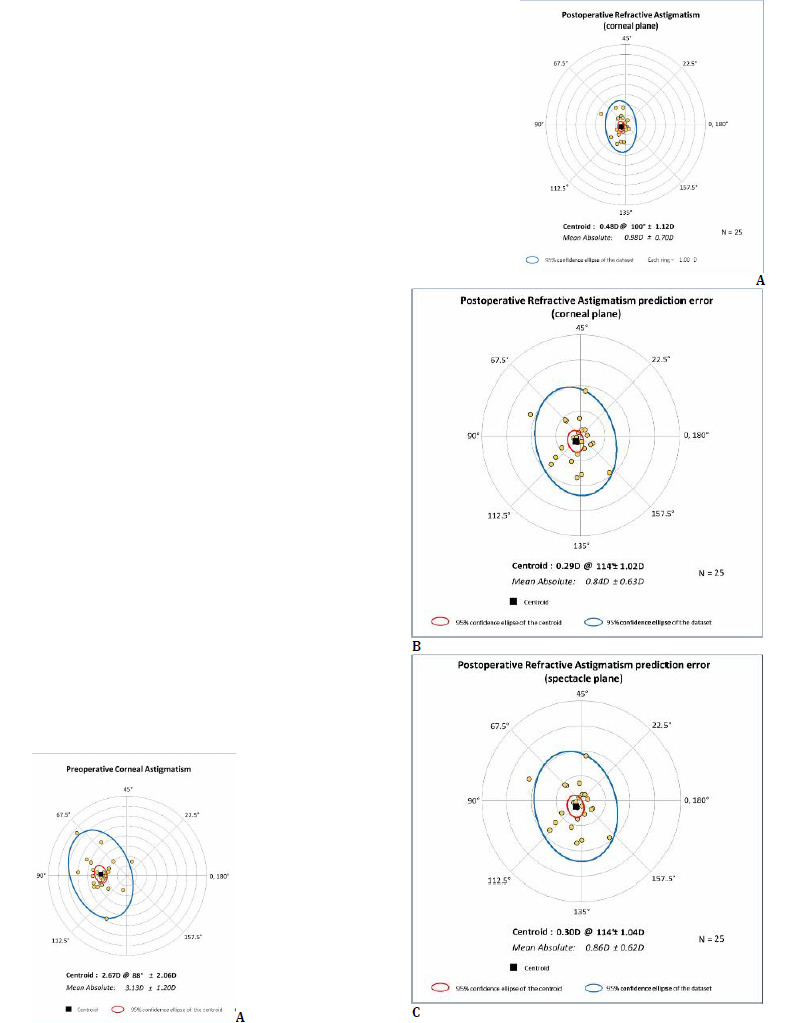
Double-angle vector plots of astigmatic outcomes following toric ICL implantation at 1 year postoperative. **A**. Preoperative corneal astigmatism (left) versus postoperative refractive astigmatism at the corneal plane. **B, C**. Postoperative refractive astigmatism prediction error at corneal plane (**B**) and at spectacle plane (**C**). Each concentric ring corresponds to 1.00 D. This figure was generated from original data using the ASCRS Astigmatism Double Angle Plot Tool (available at https://www.ascrs.org/en/tools/astigmatism-double-angle-plot-tool [23])

Double-angle plots of refractive astigmatism at the corneal plane demonstrated a marked reduction in astigmatism following toric ICL implantation (**Fig. 6A**).

The preoperative centroid was 2.67 D at 88°, with a mean absolute astigmatism of 3.13 ± 1.20 D, reflecting the cohort’s high astigmatic burden. Postoperatively, the centroid was substantially reduced to 0.48 D at 100°, with a mean absolute astigmatism of 0.98 ± 0.70 D, indicating effective astigmatic correction and a shift of the astigmatic distribution toward the origin. Postoperatively, both the magnitude of the centroid and the dispersion of data points were reduced, consistent with improved astigmatic accuracy.

Double-angle plots of prediction error demonstrated low residual astigmatism with minimal systematic bias at both the corneal (**Fig. 6B**) and spectacle planes (**Fig. 6C**). At the corneal plane, the centroid of prediction error was 0.29 D at 114°, with a mean absolute value of 0.84 ± 0.63 D. At the spectacle plane, the centroid was similarly low at 0.30 D at 114°, with a mean absolute value of 0.86 ± 0.62 D. These findings indicated a symmetric distribution of residual astigmatism around the origin and good consistency between reference planes.

### Safety outcomes

Postoperative complications are summarized in **Table 6**. At 1-month postoperatively, elevated intraocular pressure (IOP) was observed in 6 eyes (24.0%), dry eye symptoms in 3 eyes (12.0%), and dysphotopsia in 2 eyes (8.0%). The IOP rise was treated conservatively. At 1 year, no eyes had elevated IOP, while the frequency of dry eye symptoms increased: 7 eyes (28.0%) reported dry eye symptoms, scotopic-only dysphotopsia in 2 eyes (8.0%), and photopic dysphotopsia in 1 eye (4.0%). During our follow-up period, no sight-threatening complications were observed, and no eye experienced a loss of corrected distance visual acuity, cataract formation, or endothelial cell loss.

**Table 6:** Postoperative Complications Over Time (n=25 eyes)

Complication	5 days	1 month	1 year
Elevated intraocular pressure, n (%)	4 (16.0%)	6 (24.0%)	0 (0.0%)
Dry eye symptoms, n (%)	0 (0.0%)	3 (12.0%)	7 (28.0%)
Photopic Dysphotopsia, n (%)	0 (0.0%)	2 (8.0%)	1 (4.0%)
Scotopic dysphotopsia, n (%)	0 (0.0%)	0 (0.0%)	2 (8.0%)

Data are presented as numbers (percentages). N = number of eyes.

### Postoperative ICL vault

**Fig. 7:**
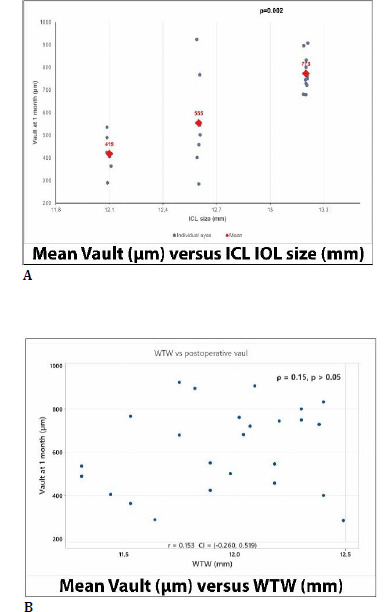
Association between mean postoperative vault versus ICL size and WTW at 1-month postoperative. **A**. Postoperative mean vault versus ICL size. Individual data points represent each eye, and red markers indicate group mean values (Kruskal-Wallis test, p=0.002). **B**. Scatterplot showing the relationship between ANTERION-derived WTW and postoperative vault. No significant correlation was observed (Spearman p=0.15, p>0.05)

Postoperative vault at 1 month averaged 615.5 ± 192.3 µm (range 286-924 µm) (**Table 7, Fig. 7**). Most eyes (68.0%) achieved vault values within the ideal range (250-750 µm), while no eyes exhibited low vault (< 250 µm). High vault (> 750 µm) was observed in 8 eyes (32.0%), all of which were implanted with 13.2 mm ICLs.

**Table 7:** Postoperative vault at 1 month and its association with ICL size and white-to-white (WTW) measurement (N = 25 eyes)

Parameter	Result
**Overall vault (µm)**	615.5 ± 192.3 (286-924) (n=25)
**Vault by ICL size (µm)**	
12.1 mm	418.7 ± 88.1 (n=6 eyes)
12.6 mm	554.8 ± 203.2 (n=8 eyes)
13.2 mm	13.2 mm: 773.4 ± 77.7 (n=11 eyes)
**Kruskal-Wallis test (vault vs ICL size)**	p = 0.002^*^
**WTW-vault correlation (Spearman)**	ρ = 0.15 (95% confidence interval -0.26 to 0.52)^†^

Data are presented as mean ± standard deviation (range) unless otherwise specified. Vault categories were defined as low (<250 µm), optimal (250–750 µm), and high (>750 µm). *Association between vault and ICL size was assessed using the Kruskal–Wallis test. †Correlation between WTW and vault was assessed using Spearman’s rank correlation.

Vault varied significantly by ICL size (Kruskal-Wallis test, p = 0.002), with a progressive increase as lens size increased. Mean vault values were 418.7 ± 88.1 µm for 12.1 mm lenses, 554.8 ± 203.2 µm for 12.6 mm lenses, and 773.4 ± 77.7 µm for 13.2 mm lenses (**Fig. 7A**). Exploratory analysis demonstrated no significant correlation between ANTERION-derived white-to-white (WTW) measurements and postoperative vault (Spearman ρ = 0.15, 95% CI -0.26 to 0.52) (**Fig. 7B**).

## Discussion

Toric implantable collamer lens (ICL) implantation demonstrated favorable visual and refractive outcomes in the present study, with significant improvements in both uncorrected and corrected distance visual acuity at 1 year (from counting fingers to 0.16 ± 0.16 and from 0.26 ± 0.21 to 0.09 ± 0.11, respectively). The efficacy (1.38 ± 0.61) and safety (1.57 ± 0.60) indices, which exceed unity, were consistent with previously reported values [7,9,24-26]. 96% of eyes achieved UDVA within one line of CDVA, and no eye lost ≥ 2 lines of CDVA. Most eyes achieved good refractive predictability. The mean SEQ was -0.56 ± 0.77 D at one year postoperatively (achieving the correction of the mean preoperative SEQ of -11.26 ± 6.62 D, attempted vs. achieved R^2^ was 0.99 with a slope of 0.96), with SEQ within ±0.50 D in 44% of eyes and within ±1.00 D in 88%. This distribution of outcomes was lower than that reported in previous toric ICL series [8,27,28] and was, in part, attributable to the inclusion of eyes with higher astigmatism. The stability analysis of the mean spherical equivalent from 1-month to 1-year postoperatively showed a slight myopic shift of -0.12 D (from -0.44 D to -0.56 D), similar to the myopization tendency reported by other authors in analyses beyond 1-year follow-up [6,8]. Regarding refractive astigmatism correction (mean preoperative refractive astigmatism of 3.72 ± 1.27 D), toric ICL implantation resulted in a mean residual astigmatism of 0.99 ± 0.72 D, with 44% of eyes within 0.50 D and 68% within 1.00 D of the target cylinder. In one case, preoperative astigmatism exceeded the maximum cylindrical correction available with current toric ICL models (outlier with preoperative refractive cylinder 8 D), resulting in a residual refractive cylinder of 2.75 D. This highlighted an inherent limitation of the technology, whereby available cylindrical power increments may not fully neutralize extreme astigmatic errors. In such cases, toric ICL implantation may be considered as part of a staged refractive strategy (bioptics), achieving a significant reduction in refractive error and improved visual function [29], even when complete correction is not feasible. Planned sequential Bioptics cases were excluded from this study cohort.

Beyond scalar refractive outcomes, vector analysis, as defined by current reporting standards [16-18], enabled a comprehensive evaluation of astigmatic correction. The close agreement between target-induced (2.59 ± 0.93 D) and surgically induced astigmatism (2.56 ± 1.18 D), with a TIA vs. SIA slope of 1.04, R^2^ = 0.68, and together with a correction index slightly below unity (0.94 ± 0.25), suggests effective and proportional astigmatic correction was achieved with high toricity ICLs in our study, consistent with previous reports implementing vectorial analysis of astigmatism outcomes with modern toric ICLs [7,25,27]. The index of success was 0.35 ± 0.27 (ideal value 0), indicating good but not complete neutralization of astigmatism. The low difference vector (of 0.84 ± 0.63 D) and favorable distribution of magnitude (72% within ±0.50 D, 84% within ±1.00 D) and angle of error (mean -5.77 ± 13.29°, with 84% within ±15°) indicated accurate and well-centered astigmatic correction consistent with recent findings [4,30]. Double-angle plot analysis further confirmed a marked reduction in astigmatism magnitude, with postoperative centroids shifting toward the origin (preoperative centroid 2.67 D at 88° and postoperatively 0.48 D at 100°, resulting in a prediction error centroid at corneal plane of 0.29 D at 114°), supporting the effectiveness of toric ICL implantation in high astigmatism, adding to the pre-existing literature findings on centroid analysis [3,7].

Postoperative vault (615.5 ± 192.3 µm, range 286-924, at 1 month) was predominantly within the optimal range (68% in the optimal range), with 32% eyes presenting higher vault and no cases of low vault observed. A significant association between vault and implanted ICL size was identified, with progressively higher vault values observed as lens diameter increased (Kruskal-Wallis p = 0.002). Notably, all high-vault cases had 13.2 mm ICLs. This finding supports the current understanding that vault is primarily determined by the degree of ICL compression within the eye and its relationship with internal anterior segment anatomy [12,13]. Previous studies have consistently shown that parameters such as angle-to-angle distance, anterior chamber width, and crystalline lens configuration play a major role in vault behavior, while oversizing relative to these dimensions is a key determinant of high vault [13,19,31], regardless of whether a toric or spherical ICL is implanted [19].

In contrast, no significant correlation was observed between white-to-white (WTW) measurements and postoperative vault (Spearman ρ = 0.15, 95% Confidence Interval -0.26 to 0.52). This finding aligns with previous reports indicating that WTW is an imperfect surrogate for internal anatomical dimensions [32,33], such as sulcus-to-sulcus or angle-to-angle distance, which are more directly relevant for ICL sizing [12,31,32]. This may partly explain the absence of a significant correlation between WTW and vault in the present study. Discrepancies between external and internal measurements may lead to suboptimal sizing and unpredictable vault outcomes, particularly in eyes with biometric values outside typical ranges [32].

Additionally, device-derived WTW measurements were significantly higher than manual caliper measurements (11.96 ± 0.35 mm vs. 11.67 ± 0.36 mm), demonstrating variability between measurement techniques (mean difference: 0.26 mm, p < 0.001), consistent with previous studies [14,15]. The relatively wide limits of agreement (-0.18 to 0.69 mm) suggest that these methods should not be used interchangeably, as this may influence ICL sizing decisions, consistent with the conclusions of previously conducted studies on WTW analysis [9,14,15]. However, given the absence of a significant association between WTW and vault in this cohort, these differences did not translate into clinically meaningful differences in vault outcomes, further emphasizing the limited predictive value of WTW alone, as previously reported [9,14].

The overall safety profile of the procedure was favorable, with no sight-threatening complications or clinically detectable loss of CDVA lines observed. Early postoperative intraocular pressure elevation (16% at 5 days, 24% at 1 month postoperatively) was transient and resolved with conservative management, with no cases by 1 year (0% cases), possibly due to the postoperative steroid eyedrops use or initial reaction of the aqueous flow dynamics to the implant [10]. Other symptoms, such as dry eye (28% at 1 year), dysphotopsia (4% at 1 year), and night vision dysphotopsia (8% at 1 year), were encountered but were clinically mild. No cataract formation or endothelial cell loss was observed during the one-year follow-up period. These findings support the established safety profile of modern posterior chamber ICL implantation with central port design [9,34].

This study had several limitations. The sample size was relatively small (N=25 eyes, 16 patients), reflecting the limited availability of patients with high astigmatism who are undergoing toric ICL implantation, a subgroup less commonly encountered in routine clinical practice. The inclusion of bilateral eyes (9 bilateral patients) may introduce inter-eye correlation; however, this design reflects real-world clinical conditions. Postoperative vault was assessed only at 1 month, precluding evaluation of long-term vault stability. In addition, postoperative ICL rotational stability was not systematically recorded, limiting further analysis of axis-related outcomes.

Despite these limitations, the study had important strengths. It provided real-world data from a relatively uncommon cohort of patients with high astigmatism and applied vector analysis in accordance with current reporting standards, enabling a comprehensive and standardized assessment of astigmatic outcomes. The combined evaluation of refractive results, vector metrics, vault behavior, and biometric measurements offered a clinically relevant and methodologically robust perspective on toric ICL implantation.

## Conclusion

Toric ICL implantation demonstrated favorable visual and refractive outcomes with a good safety profile in this cohort of patients with high astigmatism. Vector analysis confirmed accurate and proportional astigmatic correction. Postoperative vault was significantly associated with ICL size but not with white-to-white measurements, reinforcing the concept that vault is primarily determined by internal ocular anatomy rather than external biometric surrogates. These findings support the use of toric ICLs for correcting high astigmatism and highlight the need for individualized sizing strategies. Strengths of this study include standardized vector analysis and comprehensive vault evaluation in a real-world cohort that is underrepresented in the current literature.
